# A case of ulcerative colitis complicated by a simple appendiceal opening

**DOI:** 10.1002/ccr3.8573

**Published:** 2024-04-08

**Authors:** Xiao Liu, Fang Fang, Qingfeng Luo

**Affiliations:** ^1^ Department of Gastroenterology, Beijing Hospital, National Center of Gerontology, Institute of Geriatric Medicine Chinese Academy of Medical Sciences Beijing China; ^2^ Department of Pathology, Beijing Hospital, National Center of Gerontology, Institute of Geriatric Medicine Chinese Academy of Medical Sciences Beijing China

**Keywords:** appendiceal orifice, mesalazine, patient compliance, ulcerative colitis

## Abstract

This case report describes the clinical course of a 64‐year‐old male with intermittent abdominal pain attributed to recurrent ulcers at the appendiceal orifice. Initial investigations in November 2019 revealed chronic gastritis and ulcers at the appendiceal orifice, prompting consideration of ulcerative colitis (UC). The patient responded well to mesalazine therapy, experiencing relief from symptoms and improved colonoscopy findings in May 2020. Despite discontinuing medication, a recurrence of symptoms in August 2021 led to a repeat colonoscopy showing renewed ulcers. Mesalazine was reinstated, resulting in symptom resolution and improved colonoscopy findings by December 2021. However, in May 2023, a subsequent recurrence of abdominal pain and colonoscopy‐confirmed mucosal changes at the appendiceal orifice prompted reintroduction of mesalazine. The patient remains under regular monitoring on mesalazine therapy. This case highlights the challenges in managing recurrent appendiceal ulcers and the importance of long‐term therapeutic vigilance in suspected UC cases.

## 1 INTRODUCTION

Ulcerative colitis (UC) is a chronic non‐specific inflammatory disease of the gastrointestinal tract, often originating in the rectum and progressing retrograde to involve the entire colon and the terminal ileum. Affection of the appendix opening is observed in some UC patients, although isolated involvement of the appendix in UC is rarely reported. Herein, we present a case of recurrent UC with exclusive involvement of the appendix, highlighting that clinical manifestations of UC may atypically manifest as isolated appendiceal involvement. During hematoxylin and eosin (H&E) staining, the typical pathological features of UC include mucosal Inflammation. Specifically, H&E staining reveals inflammatory cell infiltration in the mucosal layer of the colon and rectum. This could include lymphocytes, plasma cells, and neutrophils. Other typical abnormalities include crypt abscesses, ulceration, distorted crypt architecture, neutrophilic infiltration, hyperemia, thickening of the mucosal layer, pseudopolyps, and loss of Goblet cells among others. For most cases, crypt branching and irregularities in size and shape are evident, accompanied by an escalation in chronic inflammatory cells within the lamina propria.

This case report examines the intricate clinical journey of a 64‐year‐old male with intermittent abdominal pain attributed to recurrent ulcers localized at the appendiceal orifice. The patient's diagnostic odyssey began with findings of chronic gastritis and subsequent identification of appendiceal ulcers, prompting suspicion of UC. The report tracks the patient's response to mesalazine therapy, the recurrence of symptoms despite initial improvement, and the therapeutic challenges encountered in managing this unique manifestation. This case underscores the complexities in diagnosing and managing UC, especially when marked by recurrent focal lesions, emphasizing the significance of continual vigilance and individualized therapeutic approaches.

## 2 CASE HISTORY/EXAMINATION

**FIGURE 1:**
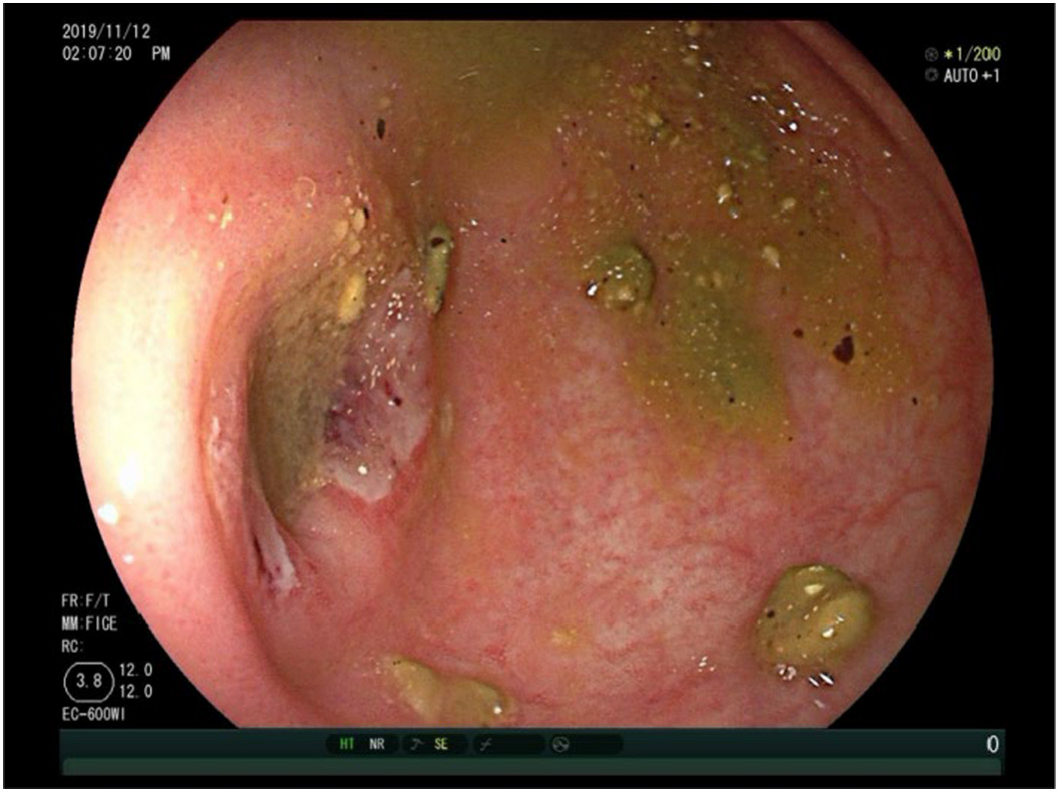
Colonoscopy in during the first patient visit: Appendiceal orifice.

**FIGURE 2:**
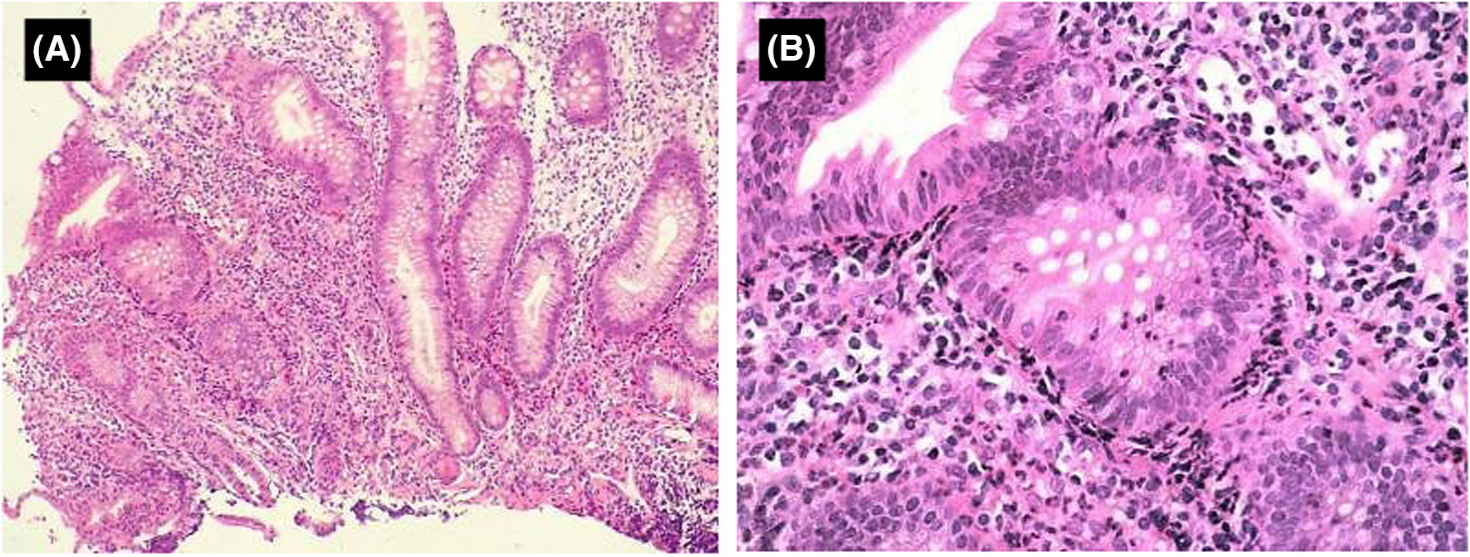
H&E staining showed moderate acute and chronic inflammation in the appendiceal orifice mucosa, accompanied by acute cryptitis. (A) 100× magnification; (B) 200× magnification.

A 64‐year‐old male presented with intermittent abdominal pain. He had a history of gastroesophageal reflux disease (GERD) but no other significant medical history. The patient initially sought medical attention due to intermittent right lower abdominal pain and sporadic loose stools. Subsequent investigations revealed chronic gastritis upon further evaluation with esophagogastroduodenoscopy, and colonoscopy indicated the presence of ulcers at the appendiceal orifice (Figure 1). There were no apparent signs of significant erythema, erosion, or ulceration in other areas of the colon. Capsule endoscopy and abdominal‐pelvic CT scans showed no notable abnormalities. Biopsy of the ulcerated appendiceal orifice during colonoscopy revealed moderate chronic mucosal inflammation with mild active changes, glandular hyperplasia, lymphoid tissue hyperplasia, and evidence of acute cryptitis (Figure 2).

## 3 METHODS (DIFFERENTIAL DIAGNOSIS, INVESTIGATION, AND TREATMENT)

**FIGURE 3:**
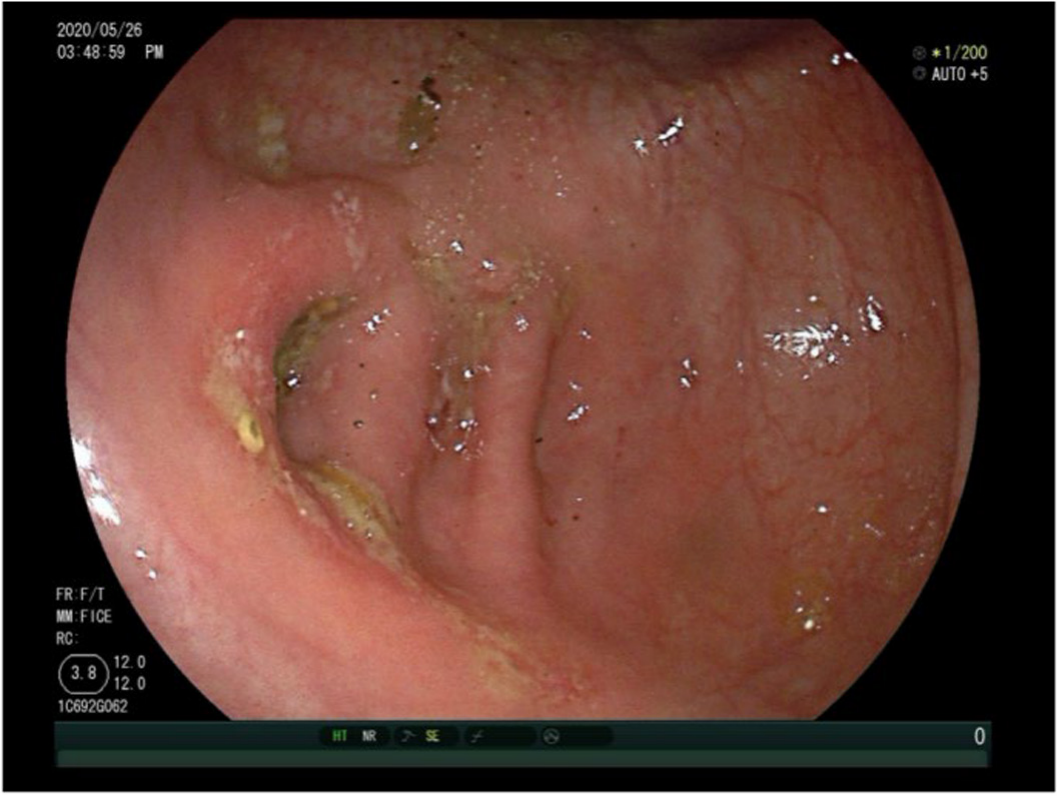
Colonoscopy 6 months after the primary office visit: Appendiceal orifice.

Considering the patient's clinical history and endoscopic findings, UC was suspected, and the patient was initiated on a daily oral dose of 3‐gram mesalazine. The patient reported gradual relief from abdominal pain, and his bowel movements became regular and formed. A follow‐up colonoscopy in 6 months post the first office visit showed improvement in the appearance of the ulcers at the appendiceal orifice, with scattered areas of congestion and erosion but no significant ulcer formation (Figure 3). Biopsy results indicated severe acute and chronic mucosal inflammation with erosions, interstitial eosinophilic infiltrates, and focal granuloma formation. The patient, feeling significantly improved, discontinued the medication on his own.

**FIGURE 4:**
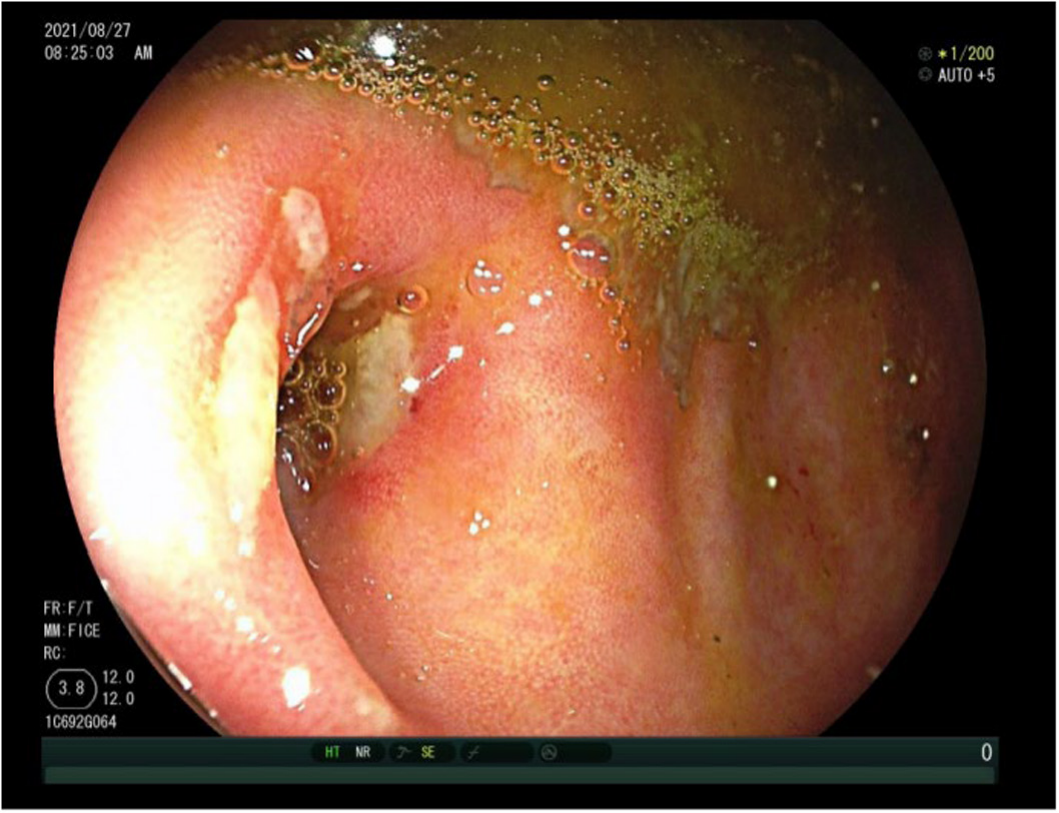
Colonoscopy conducted 21 months post the initial office visit: Examination of the appendiceal orifice.

**FIGURE 5:**
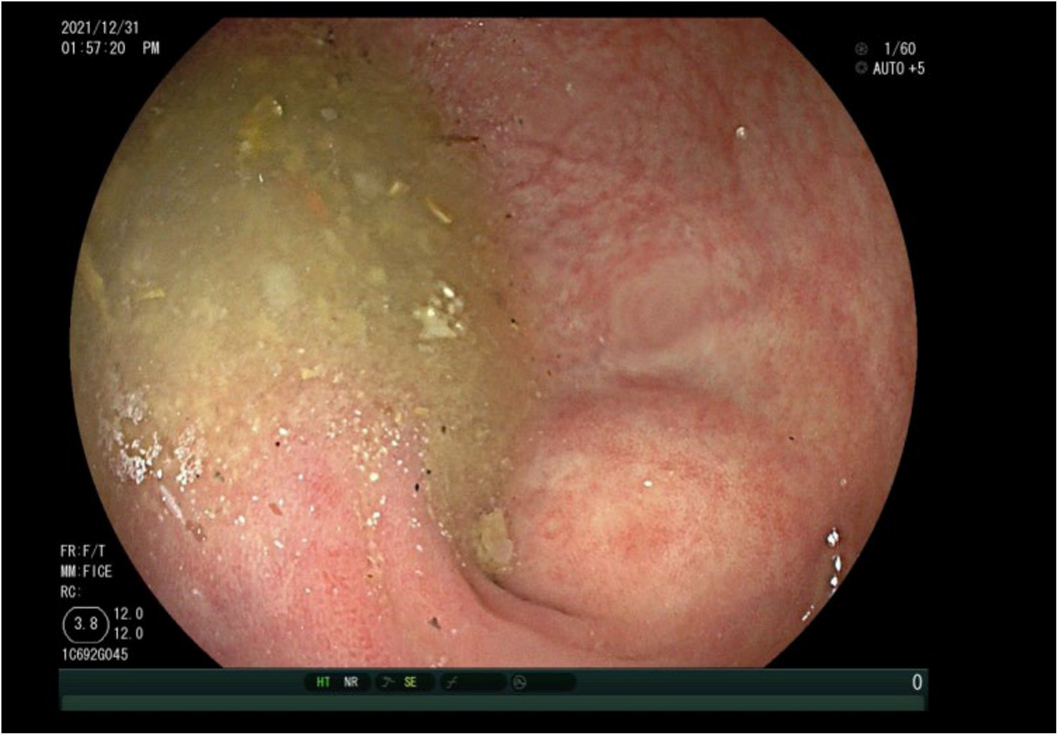
Colonoscopy conducted 2 years post the initial office visit: Examination of the appendiceal orifice.

However, 21 months after the primary office visit/diagnose, the patient experienced a recurrence of symptoms, including intermittent right lower abdominal pain, with no fever or changes in bowel habits. A repeat colonoscopy showed a recurrence of ulcers at the appendiceal orifice (Figure 4). The patient was once again treated with mesalazine, leading to symptom relief. A 2‐year follow‐up colonoscopy demonstrated a marked improvement in the appearance of the ulcers at the appendiceal orifice (Figure 5). The patient reported the disappearance of abdominal pain and continued taking mesalazine regularly for a year. The patient discontinued the use of mesalazine based on his personal decision.

## 4 CONCLUSION AND RESULTS

**FIGURE 6:**
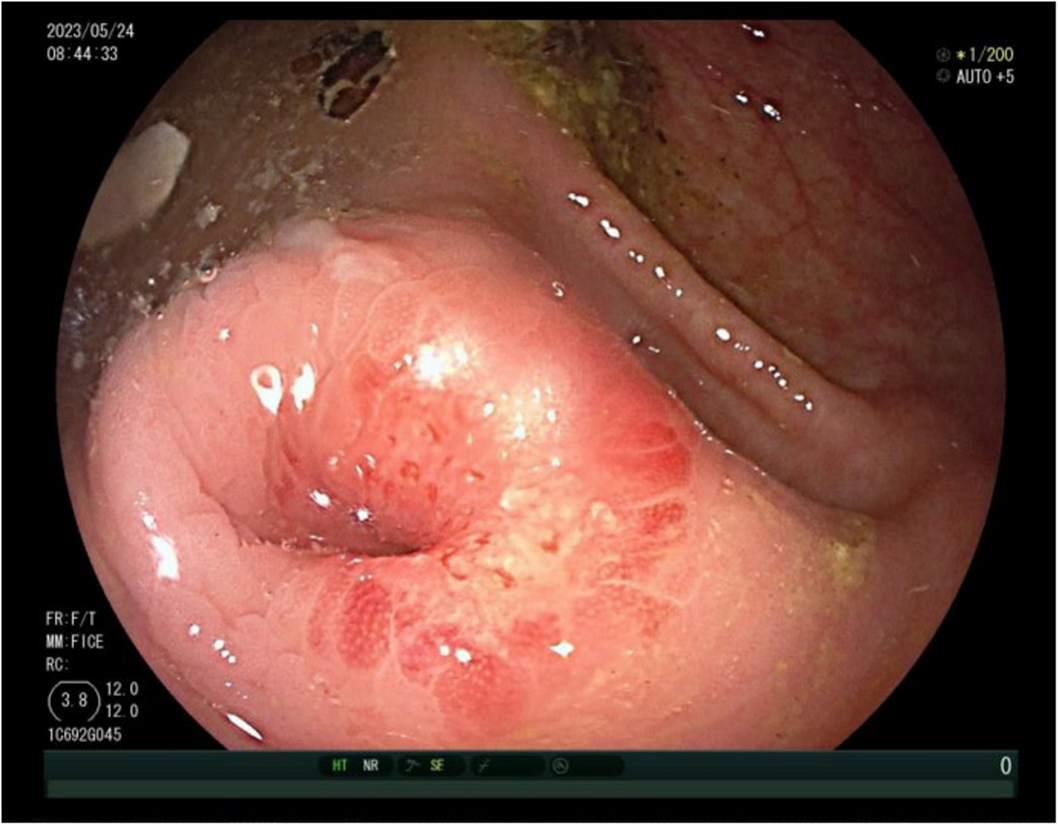
Colonoscopy conducted 42 months post the initial office visit: Examination of the appendiceal orifice.

Forty‐two months post the initial office visit, a repeat colonoscopy indicated mucosal congestion and erosion at the appendiceal orifice (Figure 6). Histopathological examination revealed acute and chronic mucosal inflammation, superficial epithelial changes with erosions, and shallow ulceration, along with evidence of cryptitis and an increased number of lamina propria plasma cells. The mucosal surfaces in other parts of the colon remained unremarkable. Mesalazine was reintroduced, resulting in the resolution of abdominal pain symptoms, and the patient continues to be regularly monitored on mesalazine therapy.

In conclusion, while UC often presents with colonic involvement, concurrent appendiceal involvement can also occur. However, there are few reports in the literature of UC solely affecting the appendix. This case demonstrates that UC can manifest as isolated appendiceal involvement. Treatment with mesalazine was effective, although continued close follow‐up with regular colonoscopies is necessary to observe disease progression.

## 5 DISCUSSION

Ulcerative colitis is a chronic non‐specific inflammatory bowel disease characterized by recurrent symptoms such as abdominal pain, diarrhea, and bloody mucous stools. The disease typically follows a chronic course with alternating periods of flare‐ups and remission, and in some cases, symptoms may persist and gradually worsen. UC usually begins in the rectum and progresses proximally, potentially affecting the entire colon, including the terminal ileum. Appendiceal orifice inflammation (AOI) refers to congestion, erosion, and ulcers at the appendiceal orifice. When AOI co‐occurs with UC, its inflammatory characteristics resemble the pathological features of UC. There is increasing evidence to suggest that AOI represents a skip lesion in the mucosa of UC.^1^ Research indicates that AOI is more common in UC patients with mild to moderate distal colonic involvement.^2^ However, retrospective studies have shown conflicting results, with some suggesting that AOI is more prevalent in moderate to severe UC.^3^ More mechanistic studies are still required.

The presence of AOI in UC patients has been associated with differing outcomes and responses to treatment. Wu and colleagues conducted a retrospective study involving 202 UC patients, of which 116 had AOI. They found a significant difference in the cumulative risk of endoscopic complete remission between the AOI and non‐AOI groups, indicating a lower rate of endoscopic complete remission in UC patients with AOI.^4^ In a long‐term follow‐up study by Oh and colleagues involving 318 UC patients, 140 of whom had AOI, the AOI group demonstrated a lower rate of endoscopic complete remission compared with the non‐AOI group. However, there were no significant differences between the two groups in terms of other clinical parameters, including the use of biologics, hospitalization rates, and proximal disease extension.^5^ Conversely, a retrospective analysis by Kyong and colleagues, involving 376 UC patients with an average follow‐up time of 66.1 months, revealed no significant differences between the AOI and non‐AOI groups in terms of endoscopic remission, hospitalization rates, recurrence rates, or the use of corticosteroids, immunosuppressants, and biologics.^6^ Therefore, the role of AOI in predicting the prognosis of UC patients remains inconclusive.

Pathologically, AOI does not possess specific characteristics. A study analyzing 26 cases with histological abnormalities at the appendiceal orifice found active inflammation in 12 cases, chronic active inflammation in 13 cases, and one case resembling collagenous colitis. Additionally, eight patients with inflammation in other biopsy samples were eventually diagnosed with UC, with none of the patients isolated to the appendiceal orifice showing clinical symptoms during follow‐up. As such, isolated inflammation of the appendiceal orifice mucosa should not be regarded as a sign of inflammatory bowel disease progression or as a distinguishing feature of other chronic colonic inflammations.^7^ Nevertheless, while AOI is not uncommon in UC patients, it is often reported as a manifestation of “skip inflammation” within UC, and cases of isolated AOI are rarely reported.

Although this case is an isolated instance, it is noteworthy due to its complete follow‐up data. The distinctive aspect lies in the manifestation of colon involvement, solely confined to the appendiceal orifice, with no abnormalities detected in other segments of the colon. The patient initially presented with isolated AOI at disease onset, exhibiting symptoms such as abdominal pain and altered bowel habits. Pathological biopsy results indicated acute cryptitis, and treatment with mesalazine resulted in significant symptom relief and improved endoscopic findings. Subsequent symptom recurrence, followed by reinitiation of treatment, also led to noticeable improvements in symptoms and endoscopic appearance. Consequently, based on symptoms, endoscopic findings, histopathology, and treatment response, the patient was diagnosed with UC, and mesalazine treatment proved effective. This case highlights that UC can occasionally present as isolated AOI without other manifestations of gastrointestinal involvement. Clinical symptom variations aligned with endoscopic changes in AOI. The patient has been followed up for over 4 years with no evidence of other colonic involvement. Future follow‐ups will monitor for potential involvement of other colonic segments.

Lastly, we also want to comment on the patient non‐compliance. While the lack of patient non‐compliance is not exclusive to UC, its repercussions are notably severe. An illustrative instance identified by the authors is a study where the remission rate exceeded 90% at 24 months among individuals adherent to mesalamine (5‐ASA), in contrast to approximately 40% in non‐compliant individuals (*p* < 0.001).^8^

## AUTHOR CONTRIBUTIONS

**Xiao Liu:** Conceptualization; data curation; formal analysis; funding acquisition; investigation; methodology; project administration; writing – original draft; writing – review and editing. **Fang Fang:** Data curation. **Qingfeng Luo:** Conceptualization.

## CONFLICT OF INTEREST STATEMENT

None declared.

## ETHICAL APPROVAL

Written informed consent was obtained from the patient.

## CONSENT

Written informed consent was obtained from the patient to publish this report in accordance with the journal's patient consent policy.

## Data Availability

Data are available on request from the authors.
